# Beneficial Effects of Probiotics on Benign Gynaecological Disorders: A Review

**DOI:** 10.3390/nu15122733

**Published:** 2023-06-13

**Authors:** Farisha Alia Norfuad, Mohd Helmy Mokhtar, Abdul Ghani Nur Azurah

**Affiliations:** 1 Department of Obstetrics and Gynaecology, Faculty of Medicine, Universiti Kebangsaan Malaysia, Kuala Lumpur 56000, Malaysia; farishaalia97@gmail.com; 2 Department of Physiology, Faculty of Medicine, Universiti Kebangsaan Malaysia, Kuala Lumpur 56000, Malaysia; helmy@ukm.edu.my

**Keywords:** probiotics, vaginal infection, polycystic ovary syndrome, endometriosis

## Abstract

Probiotics are live microorganisms that confer beneficial effects on human health when an adequate dose is administered. Recently, the use of probiotics has gained tremendous interest from the public due to its promising effects in the management of various reproductive diseases. However, the review of probiotics’ benefits on benign gynaecological disorders, including vaginal infections, polycystic ovary syndrome (PCOS) and endometriosis, remains scarce. Therefore, this review is built on current knowledge on the beneficial effects of probiotics against selected benign gynaecological disorders. Recent findings point out that probiotics’ supplementation in different clinical and in vivo models showed promising health effects and results in the amelioration of disease symptoms. Thus, in this review, we showed the findings of both studies performed in clinical settings and animal studies. However, current information, solely based on clinical trials or animal studies, is inadequate in communicating the excellent findings on the beneficial effects of probiotics on human health. Therefore, future clinical intervention studies are required to further elucidate the evidence of the benefits of probiotics benefits regarding these gynaecological disorders.

## 1. Introduction

Probiotics are defined as “living microorganisms that, when administered in sufficient amounts, confer health benefits to the host” [1]. It is important to note that probiotics is a broad term that refers to various microbes, characterised by their genus, species and strain names [2]. Today, strains classified as lactic acid bacteria are the most important in food and nutrition, and strains belonging to the genera *Lactobacillus* and *Bifidobacterium* are the most commonly used probiotics, which have essential properties in a practical context [3]. Other examples of probiotics from other genera include *Bacillus*, *Streptococcus*, *Enterococcus*, *Saccharomyces* and *Escherichia coli* (Table 1).

**Figure 1:**
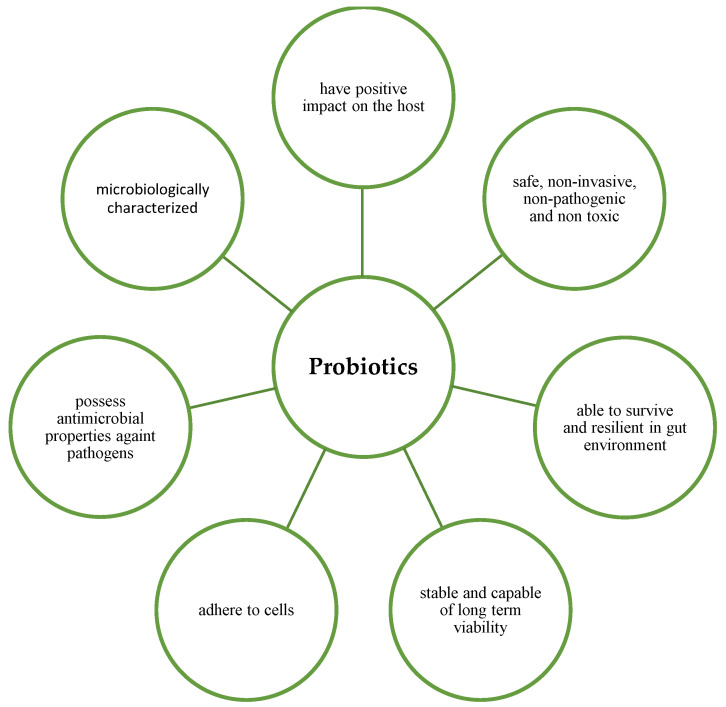
Characteristics of ideal probiotics.

A potential probiotic must possess desirable properties to exert its beneficial effects (Figure 1). According to Fuller (1989) [4], an ideal probiotic must possess certain characteristics: (1) It should be a strain that is capable of having a positive effect on the host, such as greater growth or disease resistance; (2) It should be exist in the form of living cells, preferably in large numbers, and it should be safe, non-invasive, non-pathogenic and non-toxic; (3) It should be able to survive and be resilient in the gut environment; (4) It should be stable and able to have long-term viability, i.e., survive for long periods in storage or in the field [4]. Kechagia et al. (2012) also stated that a probiotic should be of human origin, be able to adhere effectively to human intestinal cells and mucins to successfully modulate the immune system, possess antimicrobial properties against pathogenic bacteria and be microbiologically characterised. In addition, they must be tested in randomised clinical trials [5].

The sufficient dosage of probiotic microorganisms required to achieve beneficial health effects depends on the strain and the product [6]. In general, products containing probiotics should have a minimum number of viable cells with proven efficacy based on human clinical trials to transfer the effect of probiotics to the consumer. This is estimated to be between 10^6^ and 10^8^ colony-forming units per gramme (CFU/g) of the final product or 10^8^ to 10^10^ CFU/day (taking into account 100 g or 100 mL of ingested food) [7]. The rationale for administrating oral probiotics for the treatment of gynaecological disorders is related to the ability of the microorganisms to survive the gastrointestinal tract and to ascend the vaginal tract after excretion from the rectum [8]. In contrast, vaginal administration allows a direct and targeted colonisation action of probiotics to restore the balance of a normal vaginal microflora [7].

**Figure 2:**
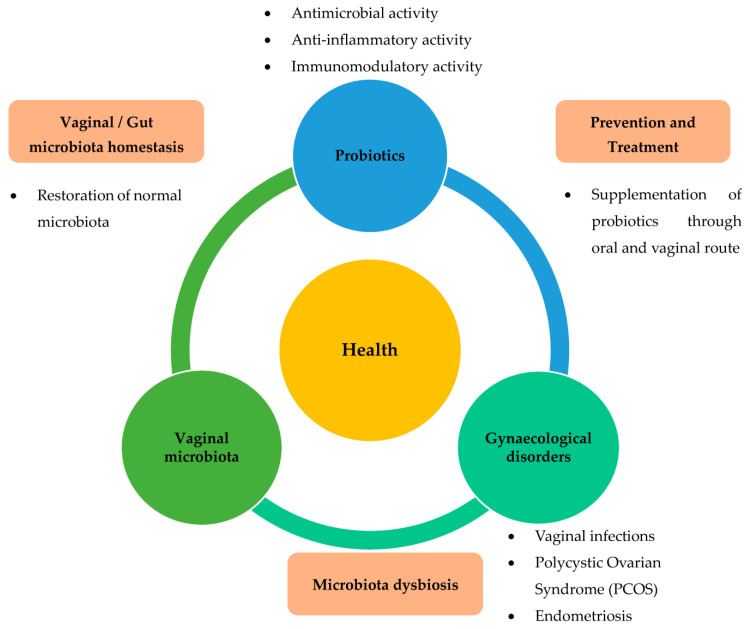
Role of probiotics relevant to gynaecological disorders via restoration of dysbiosis in the vaginal or gut microbiota. Various gynaecological disorders, such as vaginal infections, PCOS, and endometriosis, are known to be closely related to dysbiosis of vaginal or gut microbiota. Administration of probiotics has been suggested as a therapeutic approach to prevent these gynaecological diseases. Based on this information, it is now believed that there is an interplay between vaginal microbiota, probiotics, and gynaecological health and diseases.

Numerous studies have shown the positive effects of probiotics on gynaecological health in humans [9,10,11,12,13,14,15,16,17,18,19,20,21,22] and animal models [23,24]. In humans, the consumption of probiotics has been shown to be beneficial for the female reproductive system. When administered orally or vaginally to women, they are effective against gynaecological disorders, such as vaginosis, polycystic ovary syndrome, and improve the gut microbiota [25]. The administration of probiotics has been proposed as a therapeutic approach to prevent these gynaecological diseases. Based on this information, it is now believed that there is an interaction between the vaginal microbiota, probiotics, and gynaecological health and diseases (Figure 2).

Today, the consumption of probiotics to support general well-being is widespread in everyday life, not to mention the commercial success of ready-to-drink packs and various supplements in the form of sachets, tablets, and capsules. Information about their health benefits, particularly in relation to gynaecological conditions, opens the possibility of natural alternative treatments. However, there is limited evidence on health benefits of probiotics in female gynaecological disorders, such as polycystic ovarian syndrome (PCOS), dysmenorrhea, and vaginal infections. Therefore, this review examines the studies on the positive effects of probiotics on selected gynaecological disorder in women.

## 2. Methodology

A literature search was conducted to identify and evaluate relevant articles on the beneficial effects of probiotics on gynaecological conditions in women. Published, peer-reviewed and full-text articles from the period of 2000 to December 2022 were collected from electronic databases, such as Google Scholar, MEDLINE via EBSCOhost, and Scopus. The literature search was conducted using the following set of keywords: (1) Probiotics and (2) Vaginal infections or PCOS or endometriosis. Related review articles and scientific reports found from the search results were also used to further supplement the literature search.

## 3. Beneficial Effects of Probiotics on Benign Gynaecological Disorders

This section discusses the beneficial effects of probiotics based on their effects on selected gynaecological disorders in women. A total of 16 articles were discussed, comprising 6 articles on vaginal infections, 7 articles on PCOS and another 3 articles related to endometriosis.

### 3.1. Effects of Probiotics on Vaginal Infections

To date, vaginal infections are some of the most common reasons for gynaecological consultation in women of childbearing age. The most diagnosed condition is bacterial vaginosis (BV), apart from other infections, including candidiasis and trichomoniasis [26]. BV indicates aberrant alterations in the normal vaginal microbiota, due to the depletion or increased concentrations of the dominant lactic acid-producing *Lactobacilli* spp., as well as excessive proliferation of aerobic or anaerobic bacteria, normally found in the vagina [27]. BV also contributes to an increase in the local levels of pro-inflammatory cytokines and damages the epithelial and mucosal barrier. Hence, if left untreated, even the usually mild and asymptomatic BV infection can lead to an increased risk of more severe gynaecological disorders, such as endometritis, pelvic inflammatory disease, chronic vaginitis and infertility [28]. 

Probiotic therapies are emerging as popular treatment for BV. *Lactobacillus* spp. is the genus with the most well-known intravaginal beneficial probiotic species, which breaks down carbohydrates and maintains an acidic intravaginal microflora by producing lactic acid and carbon dioxide, thus preventing the growth of pathogenic microbes, such as *Enterobacteria*, *Escherichia coli*, *Candida* spp., and *G. vaginalis*, from colonising the vaginal canal [10,26,27]. Several vaginal *Lactobacillus* spp. also have protective characteristics, including the production of hydrogen peroxide and their ability to colonise the vaginal tract and adhere to the vaginal epithelial cells [29]. Apart from the characteristics of the strain, most clinical trials with positive results used probiotic formulations with high doses of *Lactobacilli* spp., about 10^9^ CFU, showing that the dosage of the administered probiotics may play a role in the success of the treatment [10,11,12,13,30,31].

Currently, the main pathogenic mechanism in BV is thought to be the loss of lactic acid-producing *Lactobacilli* spp. and the overgrowth of BV associated anaerobes such as *Gardnerella* spp., *Atopobium* spp., *Prevotella* spp., and *Mobiluncus* spp. among the vaginal microbiota [7,13]. Under these pathogenic conditions, the vaginal pH fails to be maintained at the normal range of 3.8–4.5 due to the low lactic acid level, and the hydrolytic enzymes (such as sialidase and prolidase) demolish the normal vaginal barrier, which elicits an increase in immune responses, including the release of pro-inflammatory cytokines and chemokines, such as IL-6, IL-8, IL-1α, IL-1β, TNF-α, and many others [7,32]. The synergistic interactions between pathogenic microbes increase the bacterial load and enhance the severity of BV. 

In this review, we found six articles exploring the effects of probiotics’ administration against vaginal infection in clinical trials (Table 2). These studies involved women of reproductive age or older and who had been diagnosed with a vaginal infection (bacterial vaginosis, candidiasis, trichomoniasis or combination of these conditions). The studies reported beneficial effects of probiotics against infections, mainly through a positive change in the composition of intravaginal microbiota [10,11,13]. 

Restoration of the vaginal microbiota by administration of probiotics was reported in five studies [9,12,14,32]. Ya et al. [9] recruited 120 healthy women of reproductive age with a history of BV and subjected them to BV prophylaxis with a proprietary vaginal probiotic capsule, Probaclac Vaginal, containing 10^8^ CFU of live lactic bacteria, including *L. rhamnosus*, *L. acidophilus*, *Streprococcus thermophilus* and lactose for 2 months. Throughout the treatment period, probiotic prophylaxis was reported to be successful in reducing discharge, lowering vaginal pH and dramatically reducing BV recurrence (15.8% vs. 45.0%; *p* < 0.001) and *G. vaginalis* risk in women with a history of recurrent BV. The positive outcomes of this study may be attributed to the dosing of the probiotics administered, which is 80 times higher than the amount *Lactobacillus* spp. Recommended to restore and maintain a normal intravaginal microbiota [32]. 

In another study by Ozkinay et al. [12], women with vaginal infections were recruited. A vaginal tablet containing at least 10^7^ CFU of *L. acidophilus*, 0.03 mg of estriol and 600 mg of lactose was administered daily for a period of 6 days in pre-menopausal women and for 12 days in post-menopausal women. The study reported that exogenously administered live probiotics in combination with low-dose estriol significantly improved the restoration of the vaginal microbiota. Ehrstrom et al. [14] also demonstrated for the first time that a short period of probiotics administration (5 days) can lead to a colonization of exogenous *lactobacilli* in the vaginal canal for up to 6 months. The restitution of the exogenous bacteria leads to competitive exclusion, in which serious prohibition of adhesion or displacement of pathogens takes place. The probiotics and pathogenic microbes might compete for nutrients and adhesion sites in the vaginal canal. Consequently, probiotics inhibit the development and colonization of pathogenic bacteria, viruses, or fungi by occupying these sites and utilizing the resources that are available, thus preserving a balanced microbiome and creating an inhospitable environment for the growth of pathogens [33].

Other studies confirm the influence of *Lactobacilli* spp. as an adjuvant to conventional treatment with antibiotics [10,11]. In a study by Bradshaw et al. [10], symptomatic BV-positive women were recruited and vaginal probiotic cream containing *L. acidophilus* KS400 was administered to evaluate the efficacy of probiotics as adjuvants following oral metronidazole treatment on the recurrence rate of BV. It was reported that 7 days of oral metronidazole treatment in combination with a 2% vaginal clindamycin cream, or a 12-day treatment with vaginal probiotic cream did not achieve higher cure rates for BV compared with oral metronidazole monotherapy over a six-month follow-up period. However, improved restoration of normal vaginal microbiota was demonstrated in another study by Mastromarino et al. [11], which evaluated the administration of antimicrobial metronidazole therapy for BV with oral probiotic capsules containing *L. rhamnosus* GR-1 and *L. reuteri* RC-14 for 30 days. It was found that 88% were cured in the antibiotic/probiotic group compared to 40% in the antibiotic/placebo group. 

Restoration of balanced vaginal microbiota was also reported by Vujic et al. [13] after the administration of probiotics in the form of oral capsules containing more than 10^9^ CFU *Lactobacillus rhamnosus* GR-1 and *L-reuteri* RC-14 in 243 subjects (61.52%) in the probiotic group. The restoration of a balanced vaginal microbiota was highly coincident with the presence of *lactobacilli* in the vaginal swabs, as high counts (>10^5^ CFU/mL) were found in 81.5% of subjects who received probiotics. These studies have thus shown that the overwhelming probiotic load can strongly influence the vaginal microbiota, and thus, repopulate the vagina with appropriate concentration of *Lactobacilli* sp., inhibit the growth of pathogenic microbes and prevent *G. vaginalis* and other anaerobes from adhering to the vaginal canal [9]. Probiotic strains, especially *Lactobacillus* spp., have antimicrobial properties due to their ability to produce organic acids. The repression of pathogenic growth happens because of the ability of probiotics to emit lactic acid and acetic acid. These acids lower the pH of the microbial environment and it becomes overly acidic, thus barring any pathogenic microbes that are incapable of withstanding an acidic environment [34]. 

Another important aspect of BV is the formation of polymicrobial biofilms, which are prevalent in BV samples, with *G. vaginalis* being the main component of these biofilms [35]. Studies have shown that *G. vaginalis* is able to establish a symbiotic relationship with other BV-associated anaerobes, as the initiation of biofilm formation by *G. vaginalis* allows successive species to adhere and proliferate, hence contributing to the progression of BV [28,35]. These factors collectively contribute to the ability of these bacterial species to become dominant in an environment dominated by *Lactobacilli* spp., ultimately triggering microbial dysbiosis. Therefore, the administration of exogenous *Lactobacillus* spp. can restabilise the population of *Lactobacillus* spp./*Gardenerella* spp. and help the restitution of the normal vaginal microbiota [33]. 

Probiotics have beneficial effects on vaginal infections. Although the exact cause of vaginal infections is not yet fully elucidated, most studies suggest that recurrent infections are caused by relapse rather than reinfection [29]. This suggested mechanism supports the use of probiotics to prevent the recurrence of vaginal infections, as abnormalities of the vaginal microbiota often persist in the absence of clinical symptoms. Further research is needed to clarify the mechanism by which probiotics may exert their beneficial effect on patients with vaginal infections.

### 3.2. Effects of Probiotics on PCOS

Polycystic ovarian syndrome (PCOS) is a complex gynaecological endocrine disorder with significant and diverse clinical implications, affecting reproductive functions (infertility, hyperandrogenism, hirsutism), metabolic functions (insulin resistance, type 2 diabetes mellitus, impaired glucose tolerance, adverse cardiovascular risk profiles) and psychological features (depression, increased anxiety, deterioration in quality of life) [36]. Its prevalence varies from country to country and depends on clinical and biochemical features that differ by races and age group [37,38].

Currently, the treatment for PCOS typically involves a multidimensional approach targeting different aspects of the disorders. There are various interventions that can take place to manage symptoms and improve the quality of life, such as dietary and lifestyle modifications, medications usage, and the use of in vitro fertilization (IVF), or surgical options to improve reproductive outcomes [39,40]. Although these interventions aim to address specific aspects of the condition, such as insulin resistance, hormonal imbalances, and infertility, they may not eliminate the underlying hormonal and metabolic abnormalities associated with PCOS. Hence, in this review, seven clinical trials exploring the influence of probiotics supplementation against PCOS are discussed (Table 3). Most of these studies demonstrated positive effects on the health of women with PCOS, suggesting that probiotics supplementation may be used as an alternative or complementary treatment for PCOS. The studies suggest that probiotic supplementation may influence weight loss, biomarkers of insulin resistance, and lipid profiles in PCOS patients. These beneficial effects could be mediated by host metabolism, modulation of immunological responses and reduction in systemic inflammation [37]. 

Obesity is one of the factors that may increase the risk of PCOS by promoting hyperandrogenism, hirsutism, and infertility [41]. About 40–80% of PCOS patients are overweight or obese, which increases the likelihood these women will suffer from reproductive dysfunction (irregular menstruation and infertility issues) and various psychological disorders (anxiety, sadness and bipolar disorder) [41,42]. However, some of these features of PCOS are often reversible through lifestyle modifications, such as implementing an exercise routine and nutritional supplementation, as demonstrated in studies by Ahmadi et al. and Ghanei et al. [15,20].

In a clinical trial conducted by Ahmadi et al. [15], a probiotic capsule, consisting of three viable and freeze-dried strains, *Lactobacillus acidophilus* (2 × 10^9^ CFU/g), *Lactobacillus casei* (2 × 10^9^ CFU/g), and *Bifidobacterium bifidum* (2 × 10^9^ CFU/g), was administered daily to 60 PCOS patients for 12 weeks. The study demonstrated a significant reduction in the weight and BMI of the PCOS patients after 12 weeks of supplementation with probiotics compared to the placebo. The weight loss was associated with a significant decrease in FPG, serum insulin concentrations, HOMA-IR, HOMA-B, serum triglycerides and VLDL–cholesterol. This could be due to the hypocholesterolaemia effect of the probiotics [37]. 

Another study by Ghanei et al. [20] also supports this finding, showing that 12 weeks of treatment with Cypretrone acetate and 1 × 10^9^ CFU/g of *L. acidophilus*, *Lactobacillus plantarum*, *Lactobacillus fermentum*, and *Lactobacillus gasseri* resulted in significant weight loss and reduction in BMI compared to the placebo group. These results are consistent with a previous study by Sanchez et al. [43], which indicated that treatment with *Lactobacillus rhamnosus in* conjunction with a controlled energy intake is a helpful modulator for weight management associated with obesity, in both men and women, by lowering plasma leptin concentrations directly or through changes in microbiota composition or function. However, in five clinical trials involving probiotic supplementation and PCOS patients, no change in body weight and BMI was recorded [16,17,18,19,21].

As obesity often exacerbates PCOS symptoms, the use of probiotics and a comprehensive weight-loss program have been linked with improved body composition and modest weight loss. Studies suggest that PCOS patients are more prone to metabolic disturbances, such as hyperandrogenism, increased inflammatory factors, insulin resistance and oxidative stress parameters [36]. These conditions eventually lead to a change in the overall composition of the gut microbiota, specifically causing an alteration in Alpha (α) and Beta (β) diversity. α diversity can be used to estimate the abundance of species in a microbiome, while β diversity refers to the variety of the ecological environment [44]. The decrease in α diversity and changes in β diversity in patients with PCOS may lead to changes in intestinal functions, activates the immune system, which interferes with the insulin receptor function, and increases androgen production in the ovaries, which in turn prevents normal follicle development [36]. Thus, as a result, the dysbiosis of gut microbiota occurs. Therefore, it has been suggested that the intake of probiotics may be useful in restoring the normal homeostasis of the gut microbiota. 

In a study by Nasri et al. [17], a significant decrease in serum SHBG, insulin levels, and HOMA-IR was found after 12 weeks of supplementation with *Lactobacillus acidophilus*, *Lactobacillus casei* and *Bifidobacterium bifidum* (2 ×10^9^ CFU/g each) capsules. These results are consistent with the findings of Shoaie et al. [21], who indicated that the fasting glucose and insulin resistance significantly improved in PCOS patients with metabolic syndrome after 12 weeks of probiotic supplementation. This is consistent with the findings from studies linking short-chain fatty acids, bile acid metabolism and endotoxemia, a persistent inflammatory response, with the occurrence of insulin resistance [45]. As significant imbalance in the intestinal flora has also been observed in PCOS patients [46,47], it can be speculated that the gut microbiota may be involved in the pathogenesis of PCOS by influencing insulin resistance and systemic low-grade inflammation, altering the sex hormones and other pathological mechanisms.

Rashad et al. [19] also conducted a study wherein patients with PCOS were given 10 billion CFU probiotic capsules containing *Lactobacillus delbruekii* and *Lactobacillus fermentum* twice daily for 12 weeks. It was reported that the intake of probiotics in PCOS patients led to a significant improvement in the glucose parameters, such as fasting blood glucose, compared to baseline. There was also a significant positive impact on the lipid profile (*p* < 0.05) with a significant decrease in detected inflammatory biomarkers. These changes in the lipid metabolism and insulin resistance might be attributed to the maintenance of homeostasis in the internal microbiota as probiotics restabilize dysbiosis [48]. These findings correlated with the study by Jamilian et al. [18], which also reported significant improvement in hirsutism, total testosterone, and sex hormone-binding globulin (SHBG) levels after 12 weeks of selenium and probiotics administration. Since most PCOS patients suffer from metabolic abnormalities, treatments with probiotics may provide significant relief for these patients. As demonstrated by the studies above, probiotics can decrease the production of androgens by increasing the levels of SHBG that bind and regulate free testosterone, subsequently improving hirsutism symptoms [49]. 

These findings are also consistent with those of Ghanei et al. and Shoaei et al. [20,21] who found a reduction in inflammation by IL-6 and hs-CRP, and an increase in IL-10 after 12 weeks of probiotic capsules’ administration. In addition, Tabrizi et al. [16] reported that a 12-week probiotic intake had positive effects on sex hormone-binding globulin, hs-CRP, and total antioxidant capacity. Nasri et al. [17] also reported upon probiotics’ supplementation that serum SHBG increased significantly, as well as plasma nitric oxide and serum hs-CRP. A significant decrease in serum insulin levels was also observed after treatment. 

The beneficial effects of probiotics on PCOS are summarized in this section. It is interesting to note that while probiotics show promise in PCOS management, further research is needed to establish specific strains, dosages, and treatment durations for optimal effectiveness. It should be considered as a complementary approach alongside the existing treatment modalities, as part of a comprehensive and individualized management plan for PCOS. Further studies are needed to clarify the mechanism by which probiotics exert their beneficial effects on PCOS. Overall, probiotics have been shown to have positive effects on PCOS.

### 3.3. Probiotics Effects on Endometriosis

Dysmenorrhoea, also known as painful menstruation, is a severe, painful cramping sensation in the lower abdomen that is frequently accompanied by other symptoms during the onset of menarche [50,51]. There are two types of dysmenorrhea, primary dysmenorrhea, which refers to pain with no underlying pathology in women aged 20 years and younger, and secondary dysmenorrhoea, which refers to pain with an underlying pelvic disease or pathology, such as endometriosis, in women aged over 20 years [51,52]. The reported prevalence of dysmenorrhea in women of reproductive age varies widely, ranging from 17% to 90%, with only 12% to 14% of the cases classified as severe dysmenorrhea [50].

In comparison to primary dysmenorrhoea, in secondary dysmenorrhea, the symptoms usually appear more than two years after the onset of menarche. It may occur together with other gynaecological symptoms, such as abnormal uterine bleeding. Although it often coincides with menstrual flow, the pain and discomfort that accompany this condition can have distinct characteristics and can be caused by other causes [52,53]. Endometriosis remains the most common cause and is, therefore, the most important differential diagnosis, although other disorders, such as adenomyosis, fibroids (myomas) and pelvic inflammatory diseases, may also contribute to the symptoms [54].

In this review, three studies exploring the use of probiotics in managing the symptoms of endometriosis are discussed (Table 4). The studies demonstrated positive effects on the health of patients with endometriosis, suggesting that probiotic supplementation may be an alternative to conventional treatment. It is suggested that a dysbiotic gut microbiome may contribute to a hyper-oestrogenic environment due to increasing circulating levels of oestrogen resulting from oestrogen deconjugation, which ultimately promotes the hyper-proliferation of endometrial tissue outside the uterus resulting in pelvic pain [55,56]. As endometriosis is often influenced by hormonal imbalance, the homeostasis of microbiome may contribute to reducing the excessive growth and over-proliferation of endometrial tissues [55]. In addition, the dysregulation of the immune response in the microbiome may also play an important role in the pathogenesis of endometriosis [57]. Probiotics also interact with the immune system to promote a healthy and efficient immune response. They can increase the activity of immune cells that aid in the eradication of infections, such as phagocytes and cytokines, while simultaneously stimulating the immune cells to produce antimicrobial peptides, which exhibit direct antimicrobial effects against a range of pathogens [52,55]. 

A clinical trial by Khodaverdi et al. [22] involving 37 patients with Stage 3 and 4 endometriosis (according to the rASRM) found that after consuming *Lactobacillus acidophilus*, *Lactobacillus plantarum*, *Lactobacillus fermentum* and *Lactobacillus gasseri* compared to the placebo group, there was significant improvement in mean pain score after 8 and 12 weeks of intervention, as assessed by VAS scoring [22]. Meanwhile, a study conducted by Itoh et al. [23], which included 62 patients with endometriosis, also demonstrated the beneficial effects of probiotics. The daily consumption of two OLL2809 tablets containing 100 mg of *Lactobacillus gasseri* also resulted in a significant decrease in VAS pain intensity compared to placebo [23]. Regarding this, probiotics have been shown to have anti-inflammatory properties by reducing the production of pro-inflammatory cytokine and modulating the immune responses. By reducing inflammation, the pain and discomfort felt were some of the symptoms of endometriosis that could be alleviated. Probiotics could also have impact on the gut–brain axis, a bidirectional communication network between the gut and the central nervous system [56]. It has been hypothesised that probiotics can modify the production and signalling of neurotransmitters, including those implicated in pain perception, in the gut microbiota [55]. By regulating neurotransmitter levels, probiotics may affect pain pathways and lessen pain perception in patients with endometriosis, hence affecting the VAS scoring.

This potential efficacy of probiotics supplementation may be attributed to the regulation of the immune response against endometriosis, particularly IL-12 and NK cells. In his study, Khodaverdi et al. [22] observed an increase in IL-12 concentrations and NK cells activity after administration of OLL2809 probiotics. It was concluded that a decrease in the naturally occurring killer cells appears to be related to the severity of endometriosis, while the inability of NK cells in the peritoneal cavity to clear ectopic endometrial lesions contributes to the development of endometriosis [57]. This finding is consistent with the study by Itoh et al., who reported that NK cells suppressed the development of endometriotic lesions in the group supplemented with OLL2809 tablets [23]. The tablets that contain *Lactobacillus gasseri* elicit the secretion of IL-12 from the antigen-presenting cells, resulting in the production of cytotoxic lymphocytes through the activation of NK cells and T cells [58]. Uchida et al. [24] also confirmed that the activation of NK cells promotes the regression of endometriosis lesions.

In a study by Uchida et al. [24], the therapeutic effects of *Lactobacillus* spp. were also evaluated in endometriosis rat models. After four weeks of OLL2809 probiotics administration, it was reported that the volume of induced endometriosis lesions significantly regressed in the rat models, in which two out of nine rats were completely healed after intervention. This result suggests that probiotics have beneficial effects against endometriosis by enhancing the healing of existing endometriotic lesions. 

The beneficial effects of probiotics on dysmenorrhea, specifically endometriosis, are summarised in this section. As there is limited discussion on the association between probiotics and dysmenorrhoea, further studies are needed to determine whether probiotics have a conclusive effect against dysmenorrhoea in humans and animals. Further studies involving the mechanistic investigations in which probiotics exert their beneficial effect against dysmenorrhoea are needed.

While examining the potential benefits of probiotics in improving gynaecological health, it is essential to acknowledge the existence of possible limitations and adverse effects. Probiotics have shown promise in this field; however, their effectiveness may vary, and in some cases, it did not yield the intended outcome. It is critical to understand that some individuals with sensitive digestive systems may feel gastrointestinal discomfort after consuming probiotics, including bloating, gas, or even diarrhoea [59]. Additionally, albeit rarely, there have been reported instances of probiotic strains potentially contributing to infections in patients with specific risk factors, including individual with, for example, a small colon, resulting in an impaired intestinal barrier, or those with a central venous catheter, which provides a direct pathway for microorganisms to enter the bloodstream and cause infection [60]. This is particularly relevant for individuals with weakened or compromised immune systems, as well as patients with advanced disease progression, such as cancer patients, who may be at a higher risk of infection [61]. Therefore, further clinical studies are warranted to confirm the therapeutic benefits of probiotics and balanced risks and benefits in infection-susceptible patients. Ultimately, it is essential for any individual that is considering using probiotics as a therapeutic approach to these gynaecological disorders to seek guidance from a healthcare professional. By selecting the right strain and thoroughly analysing any potential harm, the individual’s well-being and safety are prioritized.

## 4. Conclusions

Probiotics are beneficial to health and, according to human and animal studies, work towards eliminating female gynaecological disorders such as vaginal infections, PCOS, and endometriosis. However, studies on the effect of probiotics on endometriosis, especially in clinical and animal studies, are limited. Future research could focus on the benefits of probiotics on endometriosis to explain the disparity recorded among the current studies.

## Figures and Tables

**Table 1:** Microorganisms that are considered probiotics.

Microorganisms Considered Probiotics			
*Lactobacillus* Species	*Bifidobacterium* Species	Other Lactic Acid Bacteria	Non-Lactic Acid Bacteria
*L. acidophilus*	*B. adolescentis*	*Enterococcus faecalis*	*Bacillus cereus* var. *toyoi*
*L. amylovorus*	*B. animalis*	*Enterococcus faecium*	*Escherichia coli strain nissle*
*L. casei*	*B. bifidum*	*Lactococcus lactis*	*Propionibacterium freudenreichii*
*L. crispatus*	*B. breve*	*Leuconstoc mesenteroides*	*Saccharomyces cerevisiae*
*L. delbrueckii*	*B. infantis*	*Pedioccocus acidilactic*	*Saccharomyces boulardii*
*L. gallinarum*	*B. lactis*	*Sprolactobacillus inulinus*	
*L. gasseri*	*B. longum*	*Streprococcus thermophilus*	
*L. johnsonii*			
*L. paracasei*			
*L. reuteri*			
*L. rhamnosus*			

Adapted from Holzapfel et al., 2001 [3].

**Table 2:** Clinical studies related to the beneficial effects of probiotics on vaginal infections.

Population Sample	Health Condition	Probiotic Strain Administered	Dosage	Duration of Treatment	Clinical Effects/Health Parameter Modifications	References
120	History of BV	Probaclac Vaginal capsule (10^8^ CFU of *Lactobacillus rhamnosus*, *L. acidophilus*, and *Streptococcus thermophilus*)	1 vaginal capsule per treatment day	21 days (7 days on, 7 days off, 7 days on)	Probiotic prophylaxis resulted in lower recurrence rates for women with BV.	[9]
268	BV	10^7^ CFU of live *L. acidophilus* KS400 with combined use of oral metronidazole 400 mg.	Vaginal probiotic cream	12 days	Improved restoration of normal vaginal flora in BV-positive women.	[10]
125	History of BV	10^9^ *L. rhamnosus* GR-1 and *L. reuteri* RC-14 (10^5^ CFU) with combined use of oral metronidazole 500 mg.	1 oral capsule twice daily	30 days	BV cure rate is 88% compared to 40% control.	[11]
19	Vaginal infections	Vaginal tablet containing >10^7^ CFU of *L. acidophilus*, 0.03 mg of estriol and 600 mg of lactose	1 tablet daily	6 days for pre-menopausal women, 12 days for post-menopausal women	Vaginal flora was enhanced significantly by the probiotic administration in combination with low-dose estriol.	[12]
544	Vaginal infection	Oral capsules containing >10^9^ CFU *Lactobacillus rhamnosus* GR-1 and *L-reuteri* RC-14	2 capsules daily	6 weeks	Restitution to balance vaginal microbiota is reported in 243 subjects (61.52%) in the probiotic group. High counts (>10^5^ CFU/mL) of *lactobacilli* were recovered from 81.5% of subjects who received probiotics.	[13]
360	BV, vulvovaginal candidiasis	Vaginal capsules containing between 10^8^ and 10^10^ CFU of *L. gasseri* LN40, *L. fermentum* LN99, *L. casei subsp. Rhamnosus* LN113, and *P. acidilactici* LN23, after treatment	1 vaginal tablet daily	5 days	LN had a good colonization rate in The vagina of patients with BV, and women receiving LN were cured after administration.	[14]

**Table 3:** Clinical studies related to the beneficial effects of probiotics on PCOS.

Population Sample/Sample Size	Health Condition	Probiotic Strain Administered	Dosage	Duration of Treatment	Clinical Effects/Health Parameter Modifications	References
60	PCOS patients	*Lactobacillus acidophilus* (2 × 10^9^ CFU/g), *Lactobacillus casei* (2 × 10^9^ CFU/g) and *Bifidobacterium bifidum* (2 × 10^9^ CFU/g).	1 capsule daily	12 weeks	Reduction in weight and BMI. Significant reduction in FPG, serum insulin levels, serum triglycerides and VLDL–cholesterol.	[15]
60	PCOS patients	*Lactobacillus acidophilus*, *Lactobacillus casei* and *Bifidobacterium bifidum* (2 × 10^9^ CFU/g each)	1 capsule daily	12 weeks	Significantly increased serum HGB, plasma TAC and decreased serum total testosterone, CRP	[16]
60	PCOS patients	*Lactobacillus acidophilus*, *Lactobacillus casei* and *Bifidobacterium bifidum* (2 × 10^9^ CFU/g each) and 0.8 g inulin	1 capsule daily	12 weeks	Increased SHBG, plasma NO, and serum CRP. Reduction in serum insulin and total testosterone levels.	[17]
60	PCOS patients	8 × 10^9^ CFU probiotic containing *Lactobacillus acidophilus*, *Lactobacillus reuteri*, *Lactobacillus fermentum* and *Bifdobacterium bifidum and* 200 µg selenium	1 capsule daily	12 weeks	Reduced serum total testosterone, hs-CRP, hirsutism, GSH.	[18]
100	PCOS patients	10 billions probiotic capsules (*Lactobacillus delbruekii* and *Lactobacillus fermentum*)	Twice daily	12 weeks	Significant improvement to metabolic parameters: glucose, serum lipid, significant decrease in inflammatory biomarker upon supplementation with probiotics.	[19]
60	PCOS patients	1 × 10^9^ CFU/g *L. acidophilus*, *Lactobacillus plantarum*, *Lactobacillus fermentum*, and *Lactobacillus gasseri*	Two capsules daily	12 weeks	Positive effects that reduce inflammation: considerable reduction in hs-CRP, IL-6 and increased IL-10.	[20]
72	PCOS patients	*Lactobacillus casei* 7 × 10^9^ CFU/g, *Lactobacillus acidophilus* 2 × 10^9^ CFU/g, *Lactobacillus rhamnosus* 1.5 × 10^9^ CFU/g, *Lactobacillus bulgaricus* 2 × 10^8^ CFU/g, *Bifdo-bacterium breve* 2 × 10^10^ CFU/g, *Bifdobacterium longum* 7 × 10^9^ CFU/g, *Streptococcus thermophiles* 1.5 × 10^9^ CFU/g	1 capsule daily	8 weeks	Reduced glycemic index and hs-CRP, reduced serum insulin.	[21]

**Table 4:** Clinical and animal studies related to the beneficial effects of probiotics on Endometriosis.4. Limitations and adverse effects of probiotics.

Population Sample/Sample Size	Health Condition	Probiotic Strain Administered	Dosage	Duration of Treatment	Clinical Effects/Health Parameter Modifications	References
37	Endometriosis patients	10^9^ *Lactobacillus acidophilus*, *Lactobacillus plantarum*, *Lactobacillus fermentum* and *Lactobacillus gasseri*	Once daily	8 weeks	Significant improvement regarding endometriosis-associated pain.	[22]
27	Endometriosis—induced Female Wistarr-Imamichi rats	*Lactobacillus gasseri* OLL2809	40 mg	4 weeks	OLL2809 significantly enhanced the regression of endometriosis, and 2 out of 9 rats were completely healed.	[24]
62	Endometriosis patients	100 mg of *Lactobacillus gasseri* OLL2809	Two tablets daily	3 months	Decrease in VAS of pain intensity in intervention group was significantly greater than placebo.	[23]

## Data Availability

The data presented in this study are available on request from the corresponding author.
